# Does temperature and oxygen affect duration of intramarsupial development and juvenile growth in the terrestrial isopod *Porcellio scaber* (Crustacea, Malacostraca)?

**DOI:** 10.3897/zookeys.515.9353

**Published:** 2015-07-30

**Authors:** Terézia Horváthová, Andrzej Antol, Marcin Czarnoleski, Paulina Kramarz, Ulf Bauchinger, Anna Maria Labecka, Jan Kozłowski

**Affiliations:** 1 Institute of Environmental Sciences, Jagiellonian University, Gronostajowa 7, 30-387 Kraków, Poland

**Keywords:** Temperature-size rule, Oxygen, Ontogenic development, Crustacea, Oniscidea

## Abstract

According to the temperature-size rule (TSR), ectotherms developing under cold conditions experience slower growth as juveniles but reach a larger size at maturity. Whether temperature alone causes this phenomenon is unknown, but oxygen limitation can play a role in the temperature-size relationship. Oxygen may become limited under warm conditions when the resulting higher metabolism creates a greater demand for oxygen, especially in larger individuals. We examined the independent effects of oxygen concentration (10% and 22% O_2_) and temperature (15 °C and 22 °C) on duration of ontogenic development, which takes place within the maternal brood pouch (marsupium), and juvenile growth in the terrestrial isopod common rough woodlouse (*Porcellio scaber*). Individuals inside the marsupium undergo the change from the aqueous to the gaseous environment. Under hypoxia, woodlice hatched from the marsupium sooner, but their subsequent growth was not affected by the level of oxygen. Marsupial development and juvenile growth were almost three times slower at low temperature, and marsupial development was longer in larger females but only in the cold treatment. These results show that temperature and oxygen are important ecological factors affecting developmental time and that the strength of the effect likely depends on the availability of oxygen in the environment.

## Introduction

One of the most widespread patterns in biology is the temperature-size rule (TSR), which predicts slower growth and larger adult size for ectotherms growing in cold environments (Atkinson 1994). The TSR has been confirmed in more than four-fifths of studied species, including bacteria, protists, plants, invertebrates and ectothermic vertebrates (Atkinson 1994; Forster et al. 2012). Although a considerable number of the species investigated do not follow the TSR (Aguilar-Alberola and Mesquita-Joanes 2014; Kingsolver et al. 2007; Walczynska and Serra 2014) or show the reverse pattern (Diamond and Kingsolver 2010; Walters and Hassall 2006), research has been mainly directed toward finding a single mechanism that can explain the rule as well as its exceptions (Angilletta et al. 2004b; Cabanita and Atkinson 2006; Forster et al. 2012; Klok and Harrison 2013b). Since the formulation of the TSR, various adaptive and non-adaptive physiological mechanisms have been proposed (see Ray 1960 for his earlier finding). Von Bertalanffy (1960) and Perrin (1995) argued that the different temperature sensitivity of the physiological processes that affect energy uptake and utilisation may affect growth and produce the TSR (but see Angilletta and Dunham 2003). Adult body size is the product of growth rate and development time, so it has been suggested that individuals with higher temperature thresholds for development than growth should follow the TSR (van der Have and De Jong 1996; Walters and Hassall 2006; Zuo et al. 2012). Otherwise, differences in adult body size might be driven by other factors that correlate with ambient temperature, such as season length (Ejsmond et al. 2010), mortality (Angilletta et al. 2004b) or oxygen availability (Atkinson et al. 2006; Klok and Harrison 2013a). Environmental oxygen concentration has been shown to correlate with body size in aquatic amphipods, red swamp crayfish and rotifers (Bonvillain et al. 2015; Kielbasa et al. 2014; Peck and Chapelle 2003); individuals that were reared in hypoxic conditions experienced reduced growth rate and increased development time, resulting in a smaller final body size (Frazier et al. 2001; Harrison et al. 2006). The interplay between oxygen availability in the environment and the oxygen requirements of the organism, which are both dependent on thermal conditions, may explain the patterns that are consistent with the TSR (Atkinson et al. 2006; Hoefnagel and Verberk in press; Verberk et al. 2011; Walczynska et al. in press). Because the high metabolic oxygen demands of large individuals increase more rapidly at high temperatures (Verberk et al. 2011; Woods 1999), oxygen limitation is expected to be stronger under warm conditions (Chapelle and Peck 1999; Verberk and Atkinson 2013) and at later stages of ontogeny (Aguilar-Alberola and Mesquita-Joanes 2014). Consequently, these different size- and temperature-dependent oxygen requirements of ectotherms may favour a smaller body size in warm environments and a larger size in cold ones (Atkinson et al. 2006; Frazier et al. 2001; Walczynska et al. in press).

Growth rate of crustaceans is affected by the combination of internal and external factors (Hartnoll 2001). Considering the external factors, temperature and food supply are the most important drivers of variation in growth rate. Generally, lower temperature or food supply slows down growth, but the underlying proximate mechanisms are poorly understood in crustaceans (Hartnoll 2001). We used the terrestrial isopod species common rough woodlouse (*Porcellio scaber* Latreille, 1804) to examine the effect of ambient oxygen and temperature on duration of development within the maternal brood pouch (marsupium) and on juvenile growth. Our experimental approach enabled us to disentangle the independent effects of temperature and oxygen, which are otherwise correlated in nature. Terrestrial isopods use a two-stage gas exchange system in which oxygen initially dissolves in the haemolymph and is subsequently delivered to the tissues (Wright and Ting 2006), which may lead to oxygen limitation at higher temperatures (Klok et al. 2004). Furthermore, early ontogenetic development in isopods takes place in an aqueous environment inside the brood pouch (Surbida and Wright 2001) where oxygen pressure is much lower than that of the ambient air (Strathmann 1990). At later stage of intramarsupial development, individuals undergo the change from the aqueous to the gaseous environment by absorbing the marsupial fluid (Hoese and Janssen 1989). We expected that higher temperatures would decrease the development time within the marsupium and speed up juvenile growth, which is a general trend for ectotherms, but we wanted to test whether the level of oxygen moderates this thermal effect.

## Materials and methods

### Collection and maintenance of isopods

Common rough woodlice (*Porcellio scaber*) were collected in the autumn of 2013 in Kraków, Poland. Adult males and females were kept in separate plastic boxes (205 × 150 × 97 mm) in a temperature-controlled room at 15 °C (12-h day) and 8 °C (12-h night). The bottoms of the boxes were covered with wet sand and pieces of a clay pot were provided as shelter, and the animals were supplied *ad libitum* with alder and ash leaves collected from a nearby forest. After two weeks in these conditions, males (n = 1120) and females (n = 1400) were combined and transferred to new boxes for copulation and egg-laying. Fifty females and forty males were placed in each box and distributed among the experimental conditions. The photoperiod was changed to 16 h L:8 h D to initiate reproduction (McQueen and Steel 1980).

### Experimental conditions

Animals were reared in two climate chambers (15 °C and 22 °C, POL-EKO APARATURA, Sp.j., Poland), which contained two plexi-chambers (40 × 50 × 55 cm, YETI – Agencja Reklamy, Poland) with either normoxic (22%) and hypoxic (10%) conditions. This experimental set-up gave us four temperate and oxygen combinations: 15 °C and 22% oxygen, 15 °C and 10% oxygen, 22 °C and 22% oxygen, and 22 °C and 10% oxygen. Oxygen levels were regulated (ROXY-4 four channel gas regulator, Sable Systems Europe GmbH, Germany) using oxygen (normoxic) or nitrogen gas (hypoxic), and the gases were provided by Air Products Sp. z o.o., Poland. Relative humidity was maintained at 75% by a separate dew point generator for each of the four environmental conditions (DG-4, Sable Systems Europe GmbH, Germany), and temperature and relative humidity settings were confirmed with Hygrochron iButtons (Maxim/Dallas Semiconductor, USA). The relative humidity inside the rearing boxes reached 98%, which was the humidity measured in the wild colony of isopods in Kraków.

### Gravidity and parturition

Once per week, females were checked for the presence of a marsupium. Gravidity in *Porcellio scaber* is characterised by the formation of a brood pouch on the ventral side of the body, and inside the marsupium, offspring undergo twenty discrete intramarsupial stages (Milatovič et al. 2010; Wolff 2009). After hatching from marsupium, offspring undergo two postmarsupial stages, postmarsupial mancae and juveniles (Tomescu and Craciun 1987). Each individual gravid female was transferred to a separate box (52 × 48 mm, 100 ml) containing wet sand, a piece of clay pot and alder and ash leaves as food, and the boxes were checked for the presence of newborns once per week. After releasing the mancae from marsupium, females were removed from the boxes and weighed alive to the nearest 0.01 mg (XP26, Mettler Toledo, Switzerland). The duration of intramarsupial development was defined as the time between the observation of a marsupium and the observation of offspring. Newly released mancae were maintained in the box without handling for a period of nine weeks, and only leaves were added as food if necessary. One sacrificed adult conspecific was added to each box two weeks after marsupium release to facilitate the acquisition of digestive tract symbionts, which are important to the early growth and survival of juvenile woodlice (our unpublished data). At the ages of nine and thirteen weeks after leaving the marsupium, a subsample of ten juveniles from each clutch (box) was weighed alive to the nearest 0.001 mg, and mean offspring mass was calculated by dividing the combined mass by the number of offspring.

### Statistical analyses

Statistical analysis was performed with R software (R Core Team 2014), and the graphs were made using Statistica10 (StatSoft, Inc. 2011). Prior to analysis, normality and the homogeneity of variance were checked; based on the type of data female post-parturial mass and duration of marsupial development data were logarithmically and square root transformed, respectively.

The duration of marsupial development was analysed by ANCOVA with oxygen and temperature as fixed factors and the mass of the mother as numeric covariate, and all possible interactions. In total, 401 gravid females were used in the analyses (22 °C normoxia, n = 99; 22 °C hypoxia, n = 49; 15 °C normoxia, n = 153; 15 °C hypoxia n = 100). The best model was obtained following stepwise removal of all non-significant interactions (temperature × oxygen, oxygen × maternal mass and oxygen × temperature × maternal mass).

Juvenile growth was analysed with a generalised linear mixed model (GLMM); oxygen, temperature and time since leaving the marsupium were fixed factors, and box number was a random factor. Juvenile mass data were transformed with natural logarithms. In total, we analysed 369 clutches (22 °C normoxia, n = 145; 22 °C hypoxia, n = 53; 15 °C normoxia, n = 100; 15 °C hypoxia, n = 71). All non-significant interactions (temperature × oxygen, oxygen × time and oxygen × temperature × time) were removed in a stepwise manner from the model and were not included in the final analysis.

## Results

### Duration of intramarsupial development

**Figure 1.**
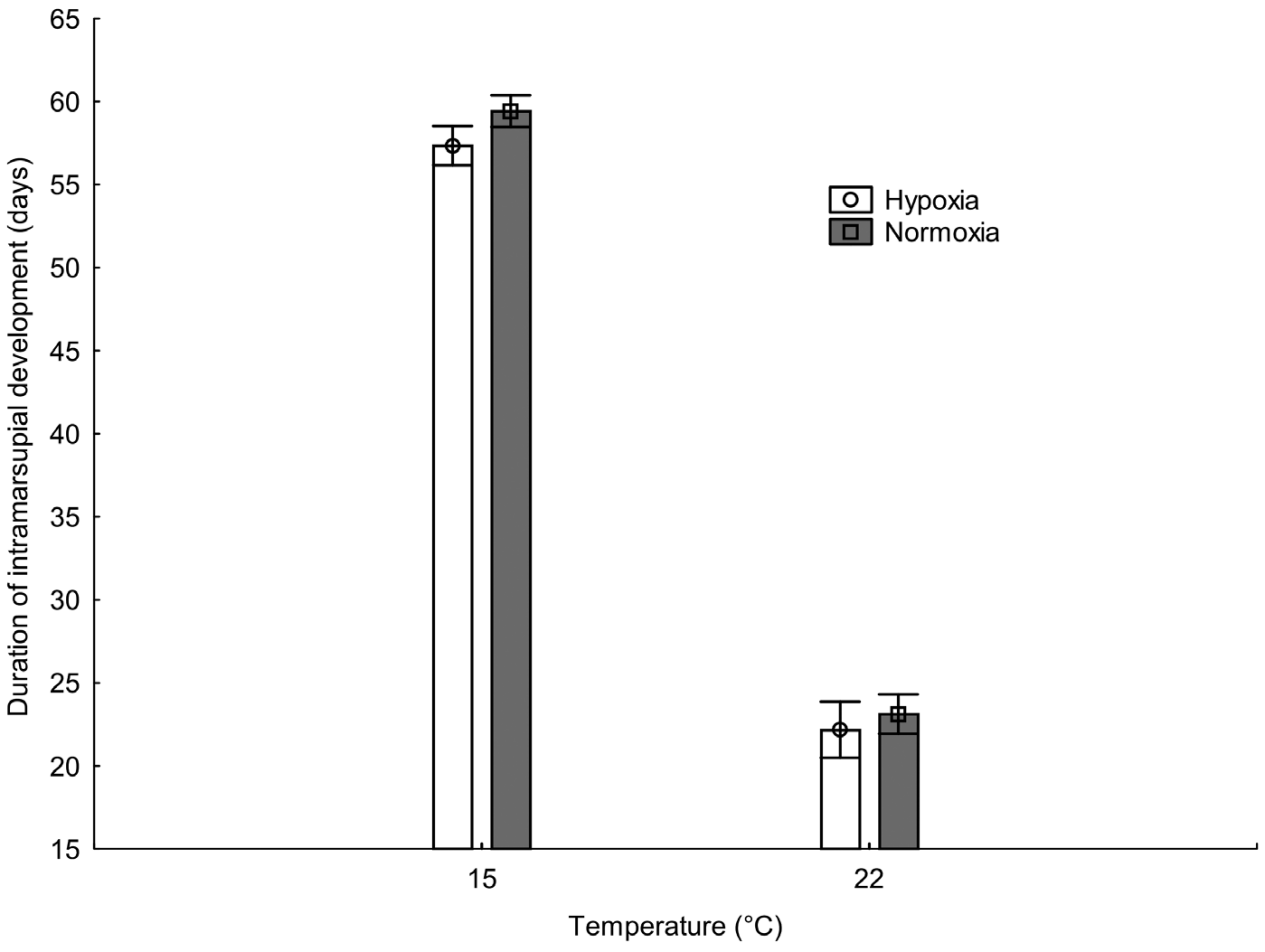
The effect of normoxia and hypoxia in cold and warm environment on the duration of intramarsupial development (expected marginal means ±CI) in the isopod *Porcellio scaber*.

**Figure 2.**
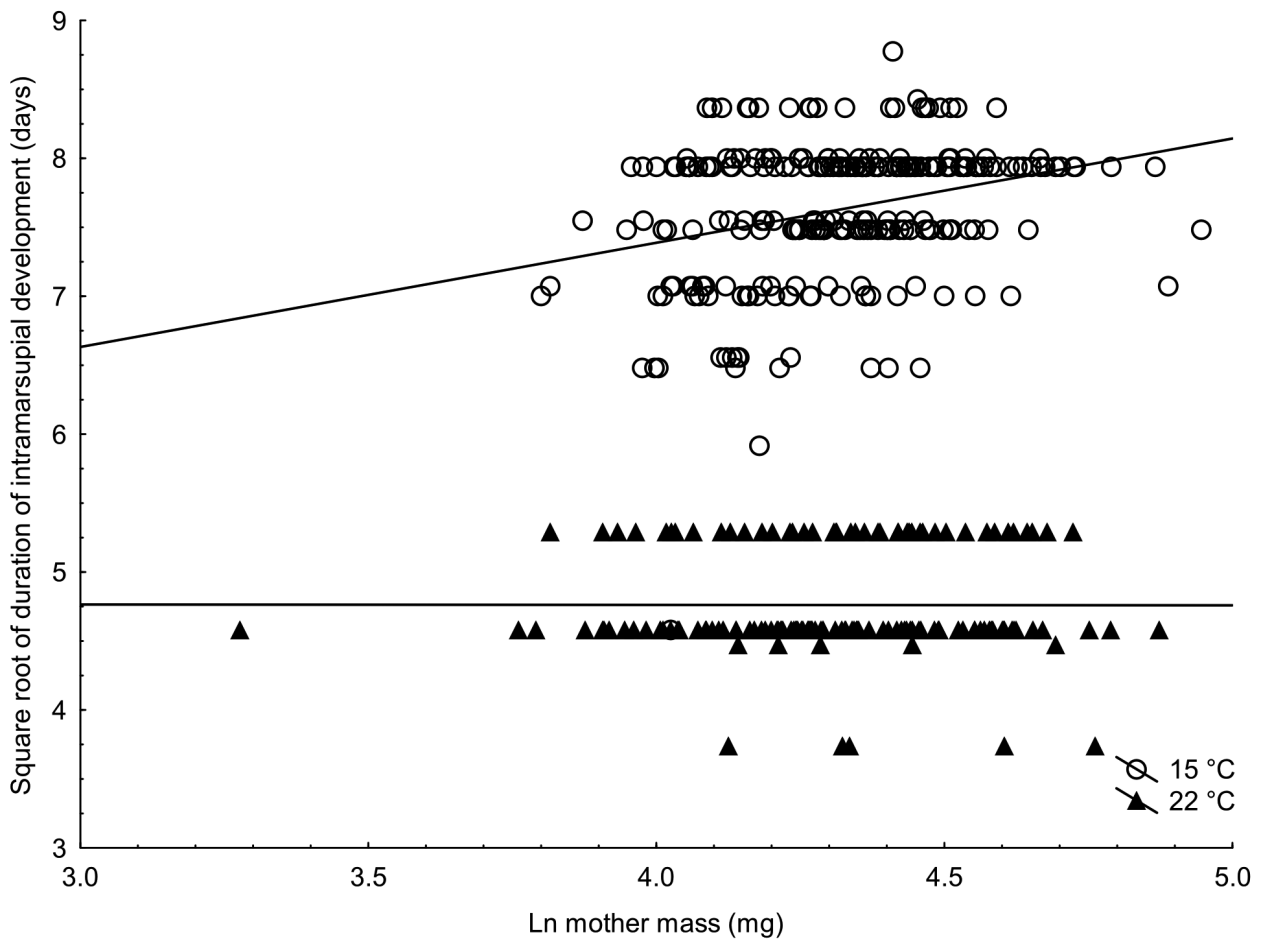
The relationship between female post-parturial mass and the duration of marsupial development in cold and warm environment in the isopod *Porcellio scaber*.

Females reared in hypoxia released their offspring from their marsupia significantly sooner than females under normoxia (15 °C: 59.3 days normoxia, 56.8 days hypoxia; 22 °C: 23.1 days normoxia, 22.4 days hypoxia; p = 0.019; Table 1, Fig. 1). Generally, the duration of intramarsupial development in warm conditions was half that in the cold temperature (23 vs. 58 days; Fig. 1), but female post-parturial mass and temperature had an interactive effect on the duration of marsupial development (p < 0.001; Table 1, Fig. 2), which caused apparent lack of significant effect of temperature (Table 1). In cold conditions, marsupial development time increased with the mass of the mother but was independent in the warm environment.

**Table 1.** Effects of temperature and oxygen on the length of marsupial development (ANCOVA) and juvenile mass (GLMM) in the isopod *Porcellio scaber*. Female post-parturial mass and juvenile mass were logarithmically transformed, and the duration of intramarsupial development was square-root transformed.

Effect	Df	F	p
**Duration of intramarsupial development**			
Temperature	1	0.3	0.568
Oxygen	1	5.6	**0.019**
Female post-parturial mass	1	28.1	**< 0.0001**
Temperature × female post-parturial mass	1	13.7	**< 0.001**
Error	393		
**Juvenile mass**			
Temperature	1	1205.2	**< 0.0001**
Oxygen	1	3.5	0.064
Time	1	2700.8	**< 0.0001**
Temperature x time	1	174.9	**< 0.0001**

### Juvenile growth

**Figure 3.**
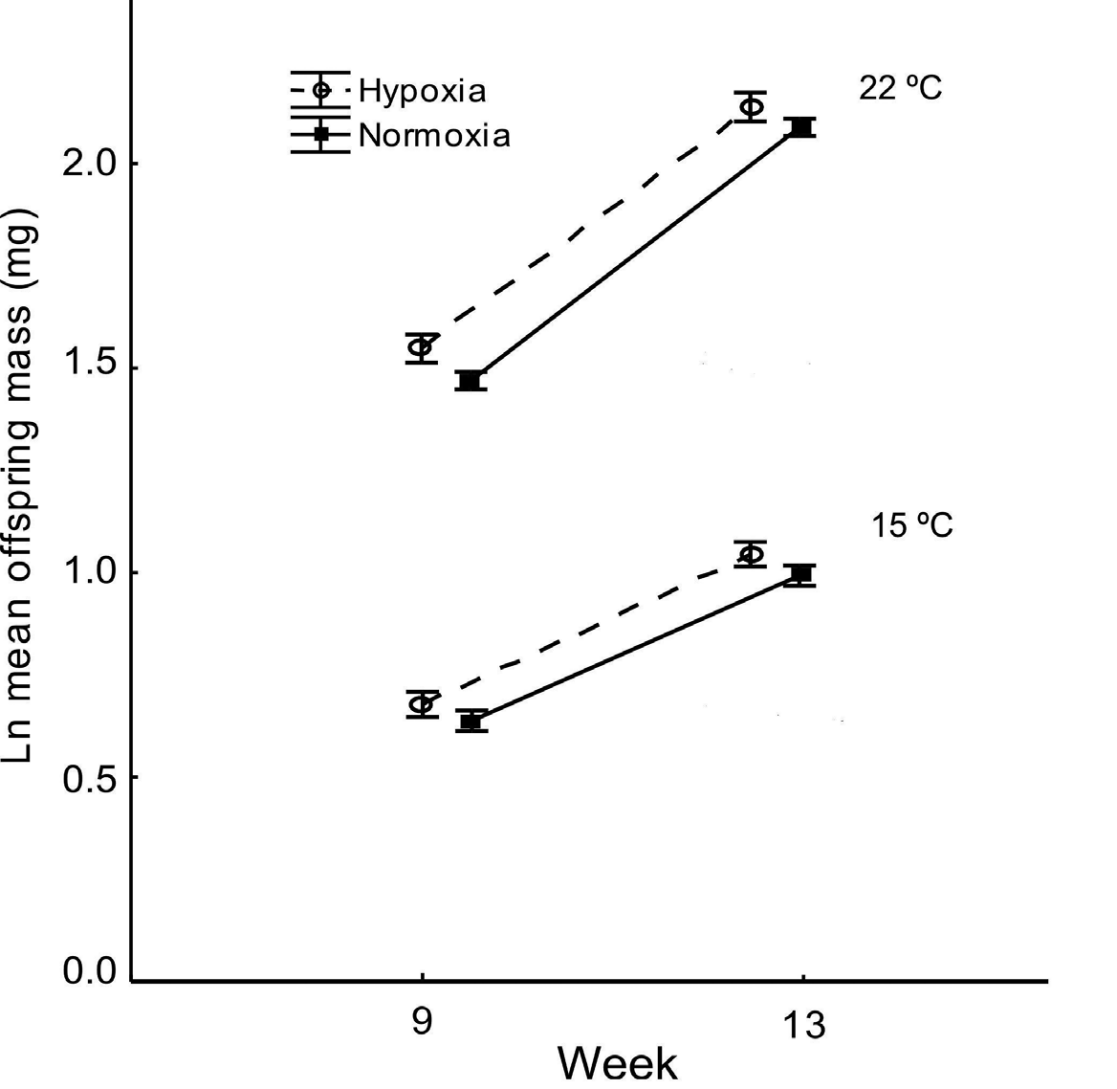
The effect of normoxia and hypoxia in cold and warm environment on juvenile growth (expected marginal means±CI) in the isopod *Porcellio scaber*.

Juveniles grew faster in warm than in cold temperatures as indicated by the significant interaction between temperature and time (p < 0.0001; Table 1, Fig. 3). Oxygen concentration did not significantly affect growth (p = 0.064; Table 1, Fig. 3).

## Discussion

We found that the duration of intramarsupial development of woodlice depended on temperature and oxygen level, with the latter effect being small, but statistically significant. Juvenile growth depended on temperature with a marginally significant effect of the level of oxygen (p = 0.064). Woodlice exposed to hypoxia completed their marsupial development sooner, and despite the shorter developmental time, juveniles under hypoxia were consistently slightly larger at both temperatures and time periods (after 9 and 13 weeks). However, because the difference was not significant, we can only conservatively state that hypoxia does not slow juvenile growth. Low temperature extended marsupial development and retarded juvenile growth; the two processes were almost two / three times slower at 15 °C than at 22 °C. Different behavioural, physiological and biochemical mechanisms may explain these patterns. For example, individuals in low temperature may just have reduced their food intake (Rombke et al. 2011), and or in alternative, low temperature may affect through decreased metabolism (Iguchi and Ikeda 2005), for example by reducing the activity of digestive enzymes as found in the mud crub *Scylla serrata* (Pavasovic et al. 2004). These results showed that temperature accelerates both development and growth whereas hypoxia shortens development time regardless of temperature.

The shortened development time under warm conditions in *Porcellio scaber* is consistent with the experimental evidence of faster development at high temperatures in a variety of crustacean species (Forster and Hirst 2012), but see Klok and Harrison (2013b), including shorter marsupial development of female Mysidacea (Crustacea) living in warm regions (Wittmann 1984). However, whether oxygen mediated this temperature response was not examined in these studies, but in accordance with the oxygen-driven TSR, we would expect smaller hatchlings under hypoxia and larger hatchlings under normoxia. Because individuals hatched earlier under hypoxic conditions, the similar body mass after nine weeks of growth in both oxygen treatments can be explained by either similar masses at hatching, which was not studied because the hatchlings were too delicate to weigh, or compensatory growth in juveniles reared in hypoxia, as observed in shrimp (*Fenneropenaeus chinensis*) (Wei et al. 2008). Because constraints on growth should arise later in ontogeny when animals are bigger and oxygen limitations are stronger (Hoefnagel and Verberk in press; Richmond et al. 2006), one could expect to find differences in growth rate at the later stages of ontogenetic development (Aguilar-Alberola and Mesquita-Joanes 2014; Forster et al. 2012). We cannot exclude that further growth until maturation would reveal such a hypothesised oxygen limitation. Therefore, applied hypoxia (10% O_2_) might be sufficient to set oxygen limits on the rate of development but not during the early growth of *Porcellio scaber* (see also Klok et al. 2004; Stevens et al. 2010).

The observed effects of differential oxygen between the rate of marsupial development on one hand and juvenile growth on the other may be related to dissimilar oxygen availability in the aqueous and gaseous environments. The early development of isopods, as well as those of other crustacean groups (e.g., Amphipoda and Mysidacea), occurs in a fluid-filled brood pouch, which protects the early stages of development against desiccation, osmotic stress and mechanical damage (Ouyang and Wright 2005; Surbida and Wright 2001). Special maternal extensions into the marsupium, called cotyledons, have been suggested to supply offspring with oxygen and nutrients (Hoese and Janssen 1989). As oxygen uptake is far more challenging in water than in air due to its higher viscosity and density (Strathmann 1990), oxygen limitations are expected to be stronger in aquatic environments (Hoefnagel and Verberk in press; Verberk et al. 2011; Walczynska et al. in press). Indeed, Forster et al. (2012) found stronger support for the TSR in aquatic than in terrestrial environments (but see Klok and Harrison 2013 for evidence of equal support). Oxygen limitations due to constraints on oxygen diffusion have mainly been found in species that carry brood pouches with tightly packed embryos (Baeza and Fernandez 2002; Fernandez et al. 2002; Lee and Strathmann 1998). If lower oxygen diffusion inside a brood pouch increases the risk of mortality, juveniles may hatch from the marsupium sooner, an effect we observe under hypoxia in both temperatures. However, we are unable to differentiate whether shorter duration of intramarsupial development in hypoxia is caused by faster developmental rate of mancae or individuals perceived hypoxia level as a stress signal and they simply left marsupium sooner. Besides the unknown cause, our results provide support that oxygen is a limiting factor in the early stages of ontogenetic development in *Porcellio scaber* that occur in the liquid phase.

The duration of intramarsupial development was not only affected by temperature and oxygen, but also by female mass; in the cold temperature, larger females incubated their progeny longer. Longer marsupial development in larger females agrees with findings for other species of terrestrial isopods: *Armadillidium vulgare*, *Cylisticus convexus* and *Porcellio scaber* (Hatchett 1947). In contrast, a negative correlation between female mass and incubation period was found in *Porcellio laevis* (Lardies et al. 2004). Because embryonic development takes place in a maternal brood pouch, its length might not only be affected by environmental factors but also by the female (i.e., a maternal effect Mousseau and Fox 1998). Females can adopt different strategies in cold and warm environments to increase the fitness of their offspring and their future prospects for reproduction (Angilletta et al. 2004a). A shorter activity window in cold environments may limit the reproductive opportunities for females (Adolph and Porter 1996), so smaller females that produce relatively smaller clutches may increase their reproductive activity by accelerating embryonic development and producing additional clutches (for different isopod species see Warburg 2013). Untouched by the effect of female mass on subsequent juvenile growth and its possible explanation, this study demonstrates that maternal factors must be considered to be of general importance when determining if animals follow the TSR.

## Conclusion

Our data show that oxygen level affects duration of intramarsupial development of the terrestrial isopod *Porcellio scaber* in an unexpected way; development is shorter under lower levels of oxygen. Although we cannot exclude the possibility that mancae hatched sooner at earlier developmental stage compared to mancae in normoxia, our results suggest that oxygen availability is crucial for development in marsupium, and future studies may be directed towards determining the developmental stages of freshly hatched mancae reared in different experimental conditions. Our results further suggest that oxygen level rather does not affect growth rate after hatching. The size of the mother may affect the rate of embryonic development to some extent, but that effect depends on the thermal environment. Duration of intramarsupial development and early growth rate are accelerated in warm compared to cold environment. We might expect that such a strong effect on early life stages may have important consequences for subsequent life stages. To what extent our observed patterns may explain life-history strategies employed by terrestrial isopods living in different thermal environments and how this in turn may affect their range expansion and geographical distribution may provide interesting approach for future investigations.
